# Whole-Body Offline-to-Online Planning for Robust Jumping of Full-Sized Humanoid Robot

**DOI:** 10.3390/biomimetics11070484

**Published:** 2026-07-10

**Authors:** Weiping Yang, Wei Zhang, Qiang Tang, Hongyang Liu, Kexin Dang, Shanchao Yan, Mingguo Zhao, Bonan Yuan

**Affiliations:** 1 AVIC Xi’an Flight Automatic Control Research Institute, Xi’an 710076, China; 2 Shenzhen International Graduate School, Tsinghua University, Shenzhen 518055, China; 3 Department of Automation, Tsinghua University, Beijing 100084, China

**Keywords:** jumping control, full-sized humanoid robot, whole-body control, offline-to-online motion planning, centroidal dynamics

## Abstract

Dynamic bipedal jumping is highly sensitive to takeoff momentum and landing configuration, making the direct execution of purely offline-optimized trajectories unreliable on full-sized humanoid robots in the presence of modeling inaccuracies and execution uncertainties. A key challenge is the gap between dynamic feasibility predicted offline using simplified models and executability on physical hardware, because such models cannot fully capture full-body dynamics, actuator behavior, contact transitions, and execution uncertainty. This paper proposes an offline-to-online planning and whole-body control framework for robust in-place jumping of full-sized humanoid robots. The framework integrates phase-consistent offline trajectory optimization, lightweight online reference reshaping, constraint-aware whole-body control, and actuator-level command mapping to improve execution robustness without online re-optimization. In the offline stage, centroidal-dynamics-based trajectory optimization generates jumping references subject to kinematic-consistency and contact-feasibility constraints. During execution, these references are adapted online using real-time state estimates to compensate for takeoff deviations and regulate the landing state; a weighted quadratic-programming whole-body controller then tracks the adapted references. Hardware experiments on a 79.5 kg humanoid robot demonstrate repeatable in-place vertical jumps with a height of approximately 30 cm and stable landings. The results show that robust jumping on a full-sized humanoid robot can be achieved by combining offline nominal trajectory generation with online execution adaptation rather than relying on exact reproduction of the offline trajectories.

## 1. Introduction

Highly dynamic motions have long been a central topic in bipedal robotics, and dynamic jumping is among the most fundamental yet challenging capabilities. Compared with walking or running, jumping places substantially greater demands on actuator power density, bandwidth, and coordination. It also involves hybrid contact transitions and a flight phase during which the robot cannot regulate its motion through ground-reaction forces. Landing typically produces large impact forces, further increasing the system’s sensitivity to modeling errors, state-estimation inaccuracies, and execution delays.

Recent public demonstrations on humanoid robot platforms, such as Boston Dynamics Atlas and Unitree H1/G1, have shown impressive dynamic motion capabilities [1,2,3,4]. However, because the detailed planning and control methods are generally not publicly available, these demonstrations are difficult to use as reproducible baselines for method-level comparison. Therefore, developing reproducible and hardware-executable planning and control frameworks for dynamic jumping remains an important research problem.

A critical difficulty of dynamic jumping arises during the takeoff phase, where body posture and centroidal angular momentum must be configured within a very short time interval. Nonzero centroidal angular momentum induces rotational motion about the center of mass, and improper takeoff configurations can therefore lead to irreversible attitude deviations during flight and unstable or failed landings [5]. Once airborne, the robot is subject to no external wrenches, and both angular momentum and horizontal linear momentum are conserved. As a result, midair posture regulation relies solely on internal shape changes that modify the composite rigid-body inertia (CCRBI) [6]. For full-sized humanoid robots, however, the achievable inertia variation is inherently limited, making posture correction during flight severely constrained and causing errors accumulated during takeoff to persist and propagate into the landing phase.

Despite significant progress in centroidal-dynamics-based planning and whole-body control, achieving robust dynamic jumping on full-sized humanoid robots remains challenging. Offline trajectory optimization can generate dynamically feasible motions that satisfy kinematic, dynamic, and contact constraints across takeoff, flight, and landing phases, but such trajectories implicitly assume that the planned takeoff impulse can be accurately realized on real hardware. In practice, actuator bandwidth limitations and force–velocity–power coupling prevent full-sized humanoid robots from delivering the required impulse within the short takeoff duration, leading to systematic tracking lag and impulse under-realization. Due to momentum conservation during flight, these residual errors cannot be corrected in subsequent phases and often manifest as degraded posture regulation and landing robustness. These challenges motivate the development of a planning and control framework that explicitly bridges offline dynamic feasibility and online execution robustness under realistic actuation constraints.

### 1.1. Related Work

Learning-based methods provide a promising route for dynamic humanoid locomotion and have demonstrated impressive parkour, jumping, and sequential contact skills [7,8,9,10]. However, for full-sized heavy humanoid robots, especially those driven by hydraulic or electro-hydrostatic actuation, safe policy deployment and sim-to-real transfer remain challenging. Large mass, high inertia, landing impacts, and complex actuator dynamics increase hardware risk and model mismatch. In jumping tasks, even small deviations in the realized takeoff impulse can directly affect flight posture and landing stability, while such errors are often difficult to explicitly analyze and compensate in end-to-end policies. These challenges motivate model-based and optimization-based methods that can explicitly represent physical constraints, actuator limitations, and hardware execution errors.

Model-based planning and control methods have long dominated research on bipedal jumping. To improve computational efficiency, many early approaches relied on reduced-order or centroidal models, such as the spring-loaded inverted pendulum (SLIP) [11,12], the single rigid-body model (SRBM) [13], and divergent component of motion (DCM) formulations derived from the linear inverted pendulum model (LIPM) [14]. By significantly reducing the dimensionality of the full-body dynamics, these methods enable real-time planning and control and have demonstrated promising results for moderate jumping and running motions [5,11,15,16]. However, the limited expressiveness of reduced-order models in capturing centroidal angular momentum regulation, inter-limb coupling, and hybrid contact dynamics makes them increasingly brittle in highly dynamic jumping scenarios. Small modeling or execution errors may accumulate into substantial attitude drift during flight, while the lack of explicit kinematic feasibility and contact wrench consistency further hinders direct deployment on full-sized humanoid hardware [5,17,18,19,20].

More recently, trajectory-optimization-based approaches have gained increasing attention as an alternative to reduced-order planning. By explicitly optimizing full-body motions across multiple phases with contact transitions, these methods incorporate kinematic reachability, dynamic consistency, and contact feasibility within a unified framework [18,21,22,23,24]. When combined with whole-body control (WBC) or model predictive control (MPC), optimized trajectories can serve as structured references that improve execution robustness under modeling uncertainty and disturbances [19,20,25,26]. Some studies have also explored the combination of offline planning and online adaptation to improve robustness against state deviations and contact uncertainties. Nevertheless, for full-sized heavy humanoid robots, large mass, high inertia, limited actuator bandwidth, and force–velocity–power constraints make it difficult to accurately realize the planned takeoff impulse on real hardware. Once systematic tracking lag or state deviation occurs during takeoff, the resulting momentum errors cannot be fully corrected during flight due to momentum conservation and directly degrade landing stability.

Overall, although offline planning with online adaptation has been explored on small-scale or lightweight platforms, robustly transferring offline feasible trajectories to real jumping motions remains challenging for full-sized heavy humanoid robots due to their large mass, high inertia, and actuator constraints. This motivates a systematic integration of offline trajectory optimization, online execution compensation, and constraint-aware whole-body control for improved hardware jumping robustness.

To further clarify the relationship between the proposed framework and existing jumping-control approaches, Table 1 provides a qualitative comparison with representative method categories. Reduced-order methods are computationally efficient but have limited model fidelity for highly dynamic full-body jumping. MPC-based methods can provide online adaptation through repeated optimization, but their computational burden increases when full-body dynamics and contact constraints are considered. RL-based methods can learn adaptive behaviors, but their performance depends heavily on training distributions and sim-to-real transfer. In contrast, the proposed framework avoids online trajectory re-optimization, retains full-body consistency through WBC, and improves landing robustness through lightweight online reshaping.

### 1.2. Overview

This paper presents an offline-to-online planning and control framework for dynamic jumping of full-sized humanoid robots under realistic actuation constraints. The core idea is to decouple offline dynamic feasibility from online execution robustness while maintaining phase consistency across takeoff, flight, and landing. Systematic takeoff execution errors are explicitly accounted for and compensated through lightweight online reference reshaping, without online re-optimization.

The framework integrates four components: (i) an offline centroidal-dynamics-based planner that generates phase-consistent jumping trajectories with kinematic and contact feasibility; (ii) a trajectory interpolator that produces time-uniform references for high-frequency control; (iii) a reshaping module that reshapes references online to compensate for impulse under-realization and regulate landing conditions; and (iv) a weighted quadratic-programming whole-body controller with actuator-level mapping for electro-hydrostatic actuators (EHAs). The approach is validated in simulation and hardware experiments on a 79.5 kg humanoid robot performing stable in-place vertical jumps.

The remainder of the paper is organized as follows. Section 2 describes the planning and control framework, Section 3 introduces the hardware platform, Section 4 presents experimental results, and Section 5 concludes the paper.

## 2. Planning and Control Framework

**Figure 1:**
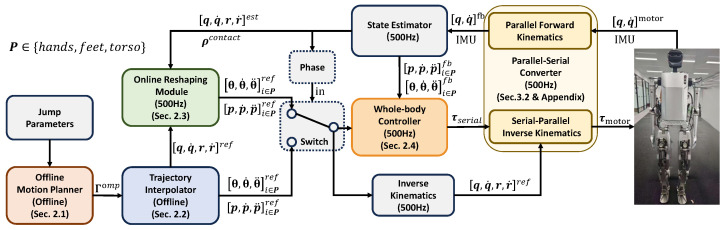
Proposed planning and control framework. (i) Given jump parameters, an offline centroidal-dynamics-based motion planner generates phase-consistent jumping trajectories, which are upsampled by a trajectory interpolator to match the high-frequency control loop. (ii) An Online reshaping module, together with phase estimation and switching logic, adapts the interpolated references online using state estimation and contact information. (iii) A weighted quadratic-programming whole-body controller tracks the selected references under full-body dynamics and contact constraints. (iv) A parallel–serial conversion module maps the serial joint-space commands to actuator-level commands for the EHA-driven joints.

In in-place bipedal jumping, systematic takeoff tracking errors caused by modeling mismatch and actuation limits cannot be corrected during the flight phase due to limited midair authority. To address this challenge, we propose an integrated planning and control framework that enforces phase-consistent offline trajectory generation while compensating takeoff execution errors through lightweight online reference adaptation and constraint-aware whole-body control. Building upon offline centroidal-dynamics-based trajectory optimization, the overall framework spans the takeoff, flight, and landing phases and is illustrated in Figure 1. State estimation is performed using an extended Kalman filter following [27].

### 2.1. Offline Motion Planner

To explicitly capture the coupling between takeoff posture and centroidal angular momentum, the jumping trajectory is generated with a centroidal-dynamics-based optimization framework [17,28]. The key modeling choice is to retain full-body kinematic consistency while enforcing only floating-base linear and angular momentum dynamics. This formulation significantly reduces the optimization dimension compared with full rigid-body dynamics, yet preserves the dynamic fidelity required for aggressive whole-body maneuvers such as takeoff and landing.

#### 2.1.1. Centroidal Model and Optimization Variables

The six-dimensional centroidal momentum is written as

(1) $$\psi_{G}=\left[\begin{array}{c} h_{G}\\ l_{G} \end{array}\right],\;\psi_{G}=A_{G}\left(q\right)\;v,$$

where $h_{G}\in R^{3}$ and $l_{G}\in R^{3}$ denote centroidal angular and linear momentum, and $A_{G}\left(q\right)$ is the centroidal momentum matrix (CMM). The generalized state is (q,v), with all vectors expressed in the world frame unless otherwise specified.

To model foot–ground interaction, the sole of each foot is discretized into *M* contact points. The centroidal dynamics are

(2) $$\begin{array}{cc} m\ddot{r} & =\sum_{i=1}^{M}F_{i}+mg, \end{array}$$

(3) $$\begin{array}{cc} \dot{h} & =\sum_{i=1}^{M}\left(c_{i}-r\right)\times F_{i}, \end{array}$$

Here, r is the CoM position, h is centroidal angular momentum about the CoM, $c_{i}$ is contact point *i*, $F_{i}={\left[F_{i,x},F_{i,y},F_{i,z}\right]}^{T}$ is the associated contact force, *m* is total robot mass, and g is gravitational acceleration. A discrete-time nonlinear program is formulated over *N* knots:

(4) $$\begin{array}{cc} \Gamma =\;\{ & q\left[k\right],v\left[k\right],r\left[k\right],\dot{r}\left[k\right],\ddot{r}\left[k\right],\\ \; & h\left[k\right],\dot{h}\left[k\right],F_{i}\left[k\right],dt\left[k\right],\;k=1,\ldots ,N\}. \end{array}$$

The generalized coordinates and velocities are defined as

(5) $$q\left[k\right]=\left[\theta_{b},p_{b},q_{j}\right]\in R^{n_{j}+7},\;v\left[k\right]=\left[\omega_{b},v_{b},{\dot{q}}_{j}\right]\in R^{n_{j}+6},$$

where $n_{j}$ is the number of actuated joints, $\theta_{b}$ is the base quaternion, and $p_{b}$ is the base position.

#### 2.1.2. Constraints Active at All Knots

For each knot k=1,…,N, the discretized centroidal dynamics and momentum consistency are imposed as

(6) $$\ddot{r}\left[k\right]=\frac{1}{m}\sum_{i=1}^{M}F_{i}\left[k\right]+g,$$

(7) $$\dot{h}\left[k\right]=\sum_{i=1}^{M}\left(c_{i}\left[k\right]-r\left[k\right]\right)\times F_{i}\left[k\right],$$

(8) $$h[k]=A_{h}\left(q\left[k\right]\right)\;v\left[k\right],$$

where $A_{h}\in R^{3\times (n_{j}+6)}$ denotes the angular block of the centroidal momentum matrix $A_{G}$. The state evolution is then enforced using a first-order discretization with variable time steps dt[k], which are included as decision variables:

(9) $$r[k]-r[k-1]=\dot{r}\left[k\right]\;dt\left[k\right],$$

(10) $$\dot{r}\left[k\right]-\dot{r}\left[k-1\right]=\ddot{r}\left[k\right]\;dt\left[k\right],$$

(11) $$h[k]-h[k-1]=\dot{h}\left[k\right]\;dt\left[k\right].$$

The kinematic consistency between the centroidal quantities, contact points, and generalized coordinates is imposed as

(12) $$r[k]=f_{\mathrm{com}}\left(q\left[k\right]\right),$$

(13) $$c_{i}\left[k\right]=f_{\mathrm{contact}}\left(q\left[k\right]\right),\;i=1,\ldots ,M,$$

(14) $$q_{j}\left[k\right]-q_{j}\left[k-1\right]={\dot{q}}_{j}\left[k\right]\;dt\left[k\right],$$

(15) $$p_{b}\left[k\right]-p_{b}\left[k-1\right]={\dot{p}}_{b}\left[k\right]\;dt\left[k\right].$$

The contact-point velocity used in stance constraints is computed from Jacobian kinematics as

(16) $${\dot{c}}_{i}\left[k\right]=J_{c_{i}}\left(q\left[k\right]\right)\;v\left[k\right].$$

The floating-base quaternion dynamics and the unit-norm constraint are given by

(17) $$\theta_{b}\left[k\right]-\theta_{b}\left[k-1\right]=\frac{1}{2}\;\Omega \left(\omega_{b}\left[k\right]\right)\;\theta_{b}\left[k-1\right]\;dt\left[k\right],$$

(18) $$∥\theta_{b}{\left[k\right]∥}^{2}=1,$$

where $\theta_{b}$ denotes the floating-base orientation quaternion, and $\Omega (\omega_{b})$ is the quaternion multiplication matrix constructed from the base angular velocity $\omega_{b}$. It is defined as

(19) $$\Omega \left(\omega_{b}\right)=\left[\begin{array}{cccc} 0 & -\omega_{x} & -\omega_{y} & -\omega_{z}\\ \omega_{x} & 0 & \omega_{z} & -\omega_{y}\\ \omega_{y} & -\omega_{z} & 0 & \omega_{x}\\ \omega_{z} & \omega_{y} & -\omega_{x} & 0 \end{array}\right].$$

To avoid degenerate time allocation in the nonlinear program, both local knot intervals and the total motion duration are bounded. The corresponding local and global time-scaling constraints are written as

(20) $$\begin{array}{c} \alpha_{\min}\frac{T}{N}\leq dt\left[k\right]\leq \alpha_{\max}\frac{T}{N}, \end{array}$$

(21) $$\begin{array}{c} \beta_{\min}T\leq \sum_{k=1}^{N}dt\left[k\right]\leq \beta_{\max}T. \end{array}$$

Here, *T* denotes the nominal horizon duration. The first inequality bounds each knot interval to avoid excessively small or large local time steps, while the second inequality constrains the total motion duration and prevents unrealistic global time scaling.

To ensure actuator feasibility, joint position and velocity limits are imposed as

(22) $$\begin{array}{c} q_{j,\min}\leq q_{j}\left[k\right]\leq q_{j,\max}, \end{array}$$

(23) $$\begin{array}{c} -v_{j,\max}\leq {\dot{q}}_{j}\left[k\right]\leq v_{j,\max}. \end{array}$$

These constraints guarantee that the optimized motion remains within hardware limits. To regularize torso posture and avoid unstable configurations, we impose

(24) $$\begin{array}{c} -{\hat{r}}_{\max}\leq \log\;\left(R^{T}\left[k\right]R_{\mathrm{ref}}\right)\leq {\hat{r}}_{\max}, \end{array}$$

(25) $$\begin{array}{c} x_{b}^{T}\left[k\right]z_{w}\leq 0. \end{array}$$

Here, R[k]∈SO(3) denotes the trunk orientation and $R_{\mathrm{ref}}$ is a reference upright posture. The logarithm map log(·) yields the minimal-axis rotation vector representing orientation error. The first inequality limits rotational deviation from the reference configuration, suppressing excessive pitch, roll, or yaw drift. The second inequality geometrically constrains the angle between the body forward axis $x_{b}\left[k\right]=R\left[k\right]e_{x}$ and the world vertical direction $z_{w}=e_{z}$, thereby preventing backward overturning.

#### 2.1.3. Phase-Dependent Constraint Activation

The horizon is partitioned into takeoff, flight, and landing:

(26) $$\begin{array}{cc} N & =N_{\mathrm{to}}+N_{\mathrm{fl}}+N_{\mathrm{ld}}, \end{array}$$

(27) $$\begin{array}{cc} K_{\mathrm{to}} & =\{1,\ldots ,N_{\mathrm{to}}\}, \end{array}$$

(28) $$\begin{array}{cc} K_{\mathrm{fl}} & =\{N_{\mathrm{to}}+1,\ldots ,N_{\mathrm{to}}+N_{\mathrm{fl}}\}, \end{array}$$

(29) $$\begin{array}{cc} K_{\mathrm{ld}} & =\{N_{\mathrm{to}}+N_{\mathrm{fl}}+1,\ldots ,N\}, \end{array}$$

and the stance index set is $K_{\mathrm{st}}=K_{\mathrm{to}}\cup K_{\mathrm{ld}}$.

For all $k\in K_{\mathrm{st}}$ (takeoff and landing), stance-contact and friction constraints are enforced:

(30) $$\begin{array}{c} c_{i,z}\left[k\right]=0,\;{\dot{c}}_{i}\left[k\right]=0, \end{array}$$

(31) $$\begin{array}{c} -\mu F_{i,z}\left[k\right]\leq F_{i,x}\left[k\right]\leq \mu F_{i,z}\left[k\right], \end{array}$$

(32) $$\begin{array}{c} -\mu F_{i,z}\left[k\right]\leq F_{i,y}\left[k\right]\leq \mu F_{i,z}\left[k\right], \end{array}$$

(33) $$\begin{array}{c} 0\leq F_{i,z}\left[k\right]\leq F_{z,\max}, \end{array}$$

where μ is friction coefficient and $F_{z,\max}$ is the normal-force limit. For all $k\in K_{\mathrm{fl}}$ (flight), contacts are deactivated:

(34) $$F_{i}\left[k\right]=0,\;i=1,\ldots ,M.$$

The CoM height is bounded during stance by

(35) $$h_{\min}\leq r_{z}\left[k\right]\leq h_{\max},\;k\in K_{\mathrm{st}},$$

where $r_{z}\left[k\right]$ denotes the vertical CoM coordinate. At the end of takeoff, the liftoff vertical speed is constrained by

(36) $$v_{z,\min}\leq {\dot{r}}_{z}\left[N_{\mathrm{to}}\right]\leq v_{z,\max}.$$

Boundary conditions enforce symmetric standing at both ends:

(37) $$q\left[1\right]=q_{\mathrm{sym}},\;q\left[N\right]=q_{\mathrm{sym}},\;v\left[1\right]=0,\;v\left[N\right]=0,$$

where $q_{\mathrm{sym}}$ is a symmetric standing posture.

#### 2.1.4. Cost Function

The offline objective is

(38) $$\begin{array}{cc} J=\sum_{k=1}^{N} & (∥q\left[k\right]-q_{\mathrm{nom}}{\left[k\right]∥}_{Q_{q}}^{2}+{∥v\left[k\right]∥}_{Q_{v}}^{2}\\ \; & +∥\ddot{r}{\left[k\right]∥}_{2}^{2}+\sum_{j=1}^{M}c_{1}{∥F_{j}\left[k\right]∥}_{2}^{2})\;dt\left[k\right]. \end{array}$$

The first two terms regularize configuration and velocity tracking with respect to a nominal trajectory $q_{\mathrm{nom}}\left[k\right]$, the third term smooths CoM acceleration, and the last term penalizes contact-force magnitude. The weighted norm is defined as ${∥x∥}_{Q}^{2}=x^{T}Qx$, with $Q_{q}⪰0$, $Q_{v}⪰0$, and $c_{1}>0$. The nominal configuration can be either a fixed conservative standing posture or a time-varying heuristic/simple-planner initialization over the full horizon.

### 2.2. Trajectory Interpolator

The offline trajectory optimization employs variable time steps to improve numerical conditioning and to allow flexible allocation of resolution across different motion phases. In contrast, the whole-body controller requires a time-uniform reference trajectory at a fixed update rate. This mismatch necessitates an explicit interpolation stage to convert the optimized trajectory into temporally consistent references suitable for high-frequency control.

In this work, the whole-body controller operates at 500Hz, and the reference trajectories are resampled at a constant time interval of 0.002s. Linear interpolation is applied to joint positions, joint velocities, and the floating-base translational position between consecutive knot points. The floating-base orientation is represented by unit quaternions and interpolated using spherical linear interpolation (slerp).

Given two consecutive orientation quaternions $p_{0}$ and $p_{1}$ at time instants $t_{0}$ and $t_{1}$, the interpolated orientation at time $t^{\prime }\in \left[t_{0},t_{1}\right]$ is computed as

(39) $$p\left(t^{\prime }\right)=\frac{\sin[(1-k)\Omega ]}{\sin\Omega }\;p_{0}+\frac{\sin(k\Omega )}{\sin\Omega }\;p_{1},\;k=\frac{t^{\prime }-t_{0}}{t_{1}-t_{0}},$$

where $\Omega =\arccos(p_{0}^{⊤}p_{1})$ denotes the angular distance between the two quaternions on the unit hypersphere. This interpolation guarantees smooth orientation evolution while preserving the unit-norm constraint.

At each control cycle, the interpolated joint states and floating-base pose are used to reconstruct the full robot configuration. Derived quantities, including the center-of-mass position, velocity, and centroidal momentum, are then recomputed from the reconstructed state. Numerical evaluation shows that the reconstructed CoM trajectory closely matches the original optimized trajectory, indicating that the interpolation process preserves the dynamic characteristics of the offline plan while providing smooth and time-consistent references for high-frequency control.

### 2.3. Online Reshaping Module

Let $x^{\mathrm{off}}\left(t\right)$ denote the offline-optimized jumping trajectory, including the CoM position and velocity, trunk pose, and foot references. When executed on full-sized humanoid robots, systematic deviations arise due to actuation limits and modeling mismatch, particularly during the short and highly dynamic takeoff phase. To mitigate these effects without online re-optimization, we introduce an online reshaping module that reshapes the offline references in a phase-consistent and low-dimensional manner.

During takeoff, insufficient realization of the planned ground-reaction impulse leads to reduced vertical CoM velocity as well as biased centroidal angular momentum at liftoff. To compensate for these systematic errors, additive acceleration terms are injected into both translational and rotational references over the takeoff phase. Specifically, the adapted CoM reference is given by

(40) $$p_{G}^{\mathrm{ref}}\left(t\right)=p_{G}^{\mathrm{off}}\left(t\right)+\int_{t_{0}}^{t}\;\int_{t_{0}}^{\tau }a_{\mathrm{cmp}}\;ds\;d\tau ,\;a_{\mathrm{cmp}}={\left[a_{x},\;0,\;a_{z}\right]}^{⊤},$$

with the corresponding velocity reference modified consistently. In addition, the trunk pitch reference θ(t) is reshaped by an additive angular acceleration,

(41) $$\theta^{\mathrm{ref}}\left(t\right)=\theta^{\mathrm{off}}\left(t\right)+\int_{t_{0}}^{t}\;\int_{t_{0}}^{\tau }a_{\mathrm{pitch}}\;ds\;d\tau ,$$

where $a_{x}$, $a_{z}$, and $a_{\mathrm{pitch}}$ are constant compensation terms applied during takeoff and bounded to preserve contact feasibility and posture limits. Importantly, the phase timing of the offline trajectory is preserved, and the compensation reshapes only the effective takeoff impulse and angular momentum without altering the motion structure.

The compensation parameters are tuned empirically based on repeated hardware experiments. Specifically, $a_{z}$ is used to compensate for the systematic under-realization of the takeoff impulse and primarily affects jump height. $a_{x}$ is used to regulate the horizontal CoM motion and landing position. $a_{\mathrm{pitch}}$ is used to compensate for takeoff-induced pitch errors and improve landing attitude stability. We observed that the jumping behavior is relatively insensitive to small variations of these parameters. The final parameter values were selected as a compromise between jump height, posture regulation, and landing robustness.

After liftoff, the robot enters a ballistic flight phase in which both linear and angular momentum are conserved. Any residual momentum generated during takeoff, therefore, directly affects landing stability. To regulate landing conditions, the nominal landing footprint is adjusted online according to the estimated CoM linear velocity $v_{G}$ and centroidal angular momentum $h_{G}$,

(42) $$\Delta r_{f}=\left[\begin{array}{c} k_{v,x}v_{G,x}+k_{h,y}h_{G,y}\\ k_{v,y}v_{G,y}+k_{h,x}h_{G,x} \end{array}\right],$$

which enables passive dissipation of residual momentum through foot placement without relying on midair force control.

Upon touchdown, the foot references are aligned with the measured contact poses, and the CoM and trunk references are smoothly guided toward a predefined standing configuration with vanishing velocities. By modifying only a small subset of translational and rotational references while preserving the offline phase structure, the proposed reshaping module provides an efficient and robust bridge between offline dynamic feasibility and real-world executability.

### 2.4. Task Setup of Whole-Body Controller

At the low-level control layer, the objective is to further improve the execution accuracy of the reference trajectories generated by the upper-level planner, while maintaining dynamical consistency and stability under multiple constraints. To this end, a weighted quadratic-programming whole-body controller is adopted. The controller can coordinate multiple control objectives while explicitly enforcing system dynamics, contact constraints, and joint-level constraints, thereby providing executable, robust, and consistent control commands for the entire robot. The whole-body controller is formulated as a Quadratic-Programming (QP) problem, where the optimization variables are defined as $x=(\ddot{q},\omega_{c})$, as shown below:

(43) $$\min_{\ddot{q},\omega_{c}}\sum_{i=1}^{N_{t}}{∥J_{i}\ddot{q}+{\dot{J}}_{i}\dot{q}-{\ddot{x}}_{i}^{des}∥}_{W_{i}}^{2}+\sum_{j=1}^{N_{c}}{∥\omega_{c}∥}_{W_{f}}^{2}$$

(44) $$s.t.\mathrm{ld}\leq H_{b}\ddot{q}+C_{b}\dot{q}+G_{b}-\sum_{j=1}^{N_{c}}J_{c_{j},b}^{⊤}\omega_{c}\leq \mathrm{ud},$$

(45) $$\omega_{c}\in C_{j},j=1,\ldots ,N_{c},$$

Here, $J_{i}$ represents the Jacobian matrix of the *i*-th task, $N_{t}$ denotes the number of tasks, and $N_{c}$ represents the number of contact points. The variable $\omega_{c}$ corresponds to the contact wrench at the foot. As observed, the *i*-th operational task is formulated as a Quadratic-Programming (QP) cost, where priority is implicitly enforced through the weight matrix $W_{i}$. The formulation of tasks and constraints follows the framework presented in [29]. The specific set of tasks and constraints employed in the jumping scenario is summarized in Table 2. Please note that the center-of-mass trajectory is not directly tracked as an explicit task; instead, it is implicitly regulated through the enforcement of floating-base dynamics and the tracking of trunk and foot motions.

## 3. System Overview

This section introduces the humanoid robot platform used to validate the proposed jumping framework, with emphasis on hardware characteristics that directly influence dynamic jumping performance. In particular, the actuator configuration and transmission mechanisms impose nontrivial force–velocity constraints, which play a critical role in explaining takeoff tracking lag and execution discrepancies observed in experiments.

### 3.1. Hardware Platform

**Figure 2:**
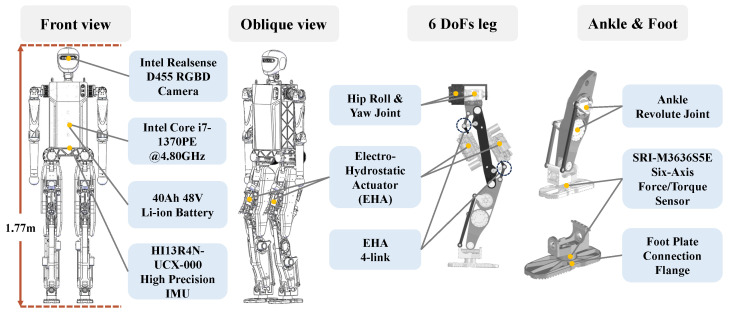
Hardware design and configuration of the bipedal humanoid robot. Each leg contains 6 dof: 3 dof for the hip joint, 1 dof for the knee joint and 2 dof for the ankle joint. Each arm contains 4 dof. Head contains 2 dof.

The humanoid robot platform shown in Figure 2 is a custom-built prototype developed to validate the proposed control framework. The robot has a total height of 1.77m and a mass of 79.5kg. Its upper body and lower limbs are vertically distributed with an approximate height ratio of 1:0.618, forming a compact morphology that is favorable for dynamic balance during jumping.

The robot is equipped with articulated upper and lower limbs. Each leg provides 6 actuated degrees of freedom for locomotion and jumping, while the two arms contribute additional degrees of freedom to assist with balance and angular momentum regulation during highly dynamic motions. A detailed summary of the robot’s physical parameters and actuator specifications is provided in Table 3.

The onboard control system is built upon a self-developed OneBox controller featuring a heterogeneous X86+GPU computing architecture, composed of a 13th-generation Intel i7 processor and an NVIDIA Jetson AGX Orin module. In the jumping experiments, the whole-body controller runs at 500Hz, corresponding to a control period of 0.002s. The interpolated reference trajectory is also updated at the same time resolution, enabling synchronized trajectory tracking and online reshaping. The online reshaping module only involves low-dimensional algebraic computations on CoM, trunk, and foot references, and therefore introduces negligible additional computational burden compared with the WBC QP. Together with trunk IMU feedback, this onboard computing architecture provides sufficient real-time capability for state estimation, contact detection, online reference compensation, and whole-body control during dynamic jumping.

Six-axis force/torque sensors are installed under both feet to measure ground-reaction forces and moments, enabling contact detection and force feedback during takeoff and landing. An industrial-grade inertial measurement unit (IMU) mounted in the trunk provides body orientation and angular velocity estimates, which are critical for monitoring centroidal angular momentum evolution. In addition, an Intel RealSense D455 depth camera is mounted on the head to support future perception-driven extensions, although vision is not directly used in the experiments reported in this paper.

### 3.2. EHA Configurations

**Figure 3:**
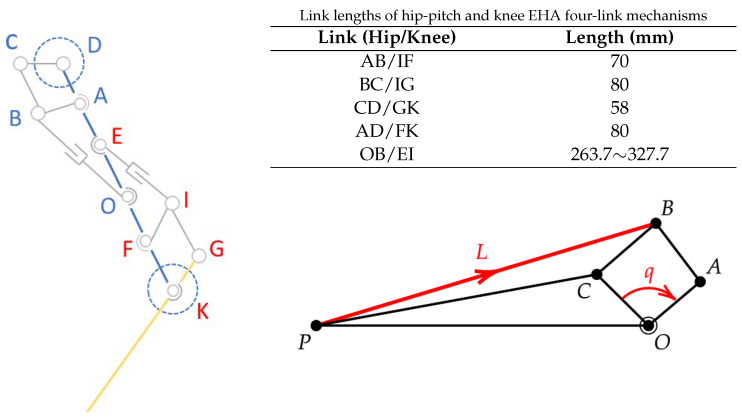
(**Left**) Schematic of the EHA-driven four-bar mechanisms. (**Top-right**) Link lengths used in the hip-pitch and knee EHA four-bar linkages. (**Bottom-right**) Geometric definition of the actuator stroke *L* and joint angle *q* in the four-link mechanism.

The hip-pitch and knee joints of the robot are actuated by electro-hydrostatic actuators (EHAs) through parallel four-bar mechanisms, as illustrated in Figure 3. This actuation scheme provides high force capability and compact mechanical integration, which are essential for generating large takeoff impulses in jumping motions. However, it also introduces a nonlinear transmission between serial joint variables used in motion planning and whole-body control, and the actuator-level quantities directly realized by the hardware.

Specifically, the planner and whole-body controller operate in joint space using joint angles *q*, angular velocities $\dot{q}$, and joint torques τ, whereas the EHA hardware is driven by actuator stroke *L*, stroke velocity $\dot{L}$, and linear output force *F*. As a result, the achievable joint torque and velocity are coupled through a configuration-dependent nonlinear transmission, which directly affects the realization of the planned takeoff impulse.

The kinematic and static relationships between joint-space variables and actuator-space quantities can be expressed as

(46) $$\dot{q}=J\left(L\right)\dot{L},\;\tau =J^{-1}\left(L\right)F,$$

where J(L) denotes the configuration-dependent transmission Jacobian of the four-bar mechanism. Due to this nonlinear mapping, actuator force and velocity limits translate into configuration-dependent joint-space constraints, which are particularly restrictive during highly dynamic phases such as takeoff and landing.

In the proposed framework, joint-space commands generated by the whole-body controller are converted to actuator-level references using this geometric mapping, enabling actuator-aware execution and monitoring. Detailed geometric derivations of the four-bar kinematics and transmission relationships are provided in the Appendix A.

Although the WBC is solved in the serial joint space, the hip-pitch and knee joints driven by EHAs are executed through actuator-level commands. At each control cycle, the actuator stroke *L* and transmission Jacobian J(L) are computed from the measured joint configuration. The WBC output $\left(q,\dot{q},\tau \right)$ is then mapped to actuator stroke, stroke velocity, and linear force using $\dot{q}=J\left(L\right)\dot{L}$ and $\tau =J^{-1}\left(L\right)F$. The resulting actuator commands are checked against the stroke, velocity, force, and power limits before being sent to the hardware, and low-level saturation limits are applied for safety. Conservative joint-space velocity, torque, and power limits are also used in the WBC to reduce the possibility of actuator saturation. No explicit model-based delay compensation is implemented; instead, execution lag is handled through the 500Hz feedback loop, online reference reshaping, and actuator-level limiting. The measured force–velocity data indicate that no sustained EHA saturation occurs in the reported jumping experiments.

## 4. Experiments

This section presents both simulation and hardware experimental results to evaluate the proposed planning and control framework. The results are organized to illustrate the feasibility of offline trajectory optimization, the execution characteristics on real hardware, and the impact of actuator-level constraints on jumping performance. The main planning and control parameters used in both simulation and hardware experiments are summarized in Table 4.

### 4.1. Offline Trajectory-Optimization Results

We first present the results of the offline trajectory optimization to demonstrate the feasibility and physical consistency of the proposed planning formulation. The high-dimensional nonlinear optimization problem was solved using the Drake toolbox [30] with the SNOPT solver [31].

**Figure 4:**
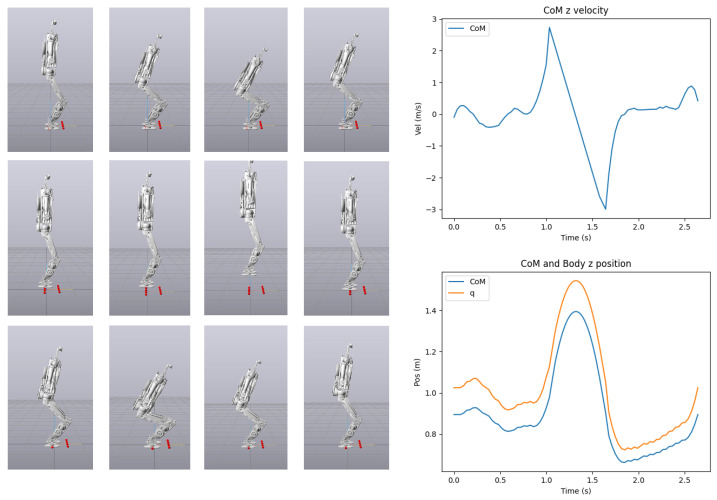
Offline trajectory optimization results for jumping motion. Left: simulated jumping snapshots, where the red markers indicate foot/contact reference markers. Right: CoM vertical velocity and CoM/base vertical positions; the colored curves denote the corresponding plotted trajectories.

During optimization, the vertical CoM velocity at liftoff is constrained within 2.0–2.4 m/s, and the CoM height at takeoff is limited to be below 0.98 m. The optimization converges in approximately 458 s. Although the computation time is relatively high, the optimization is performed entirely offline and does not affect real-time execution. The additional offline computation improves the feasibility and hardware executability of the generated trajectory by enforcing kinematic, dynamic, contact, and actuator constraints across all jumping phases. Figure 4 visualizes the optimized jumping trajectory in the Drake simulation environment. The robot autonomously generates a preparatory squatting motion, followed by rapid leg extension for liftoff, a ballistic flight phase, and a compliant squatting profile during landing for impact absorption.

These results indicate that the centroidal-dynamics-based formulation, together with task-specific kinematic and contact constraints, can generate dynamically feasible jumping motions that satisfy posture, momentum, and contact consistency requirements. The optimized trajectories therefore serve as suitable nominal references for real-robot execution.

### 4.2. Jumping Simulation

**Figure 5:**
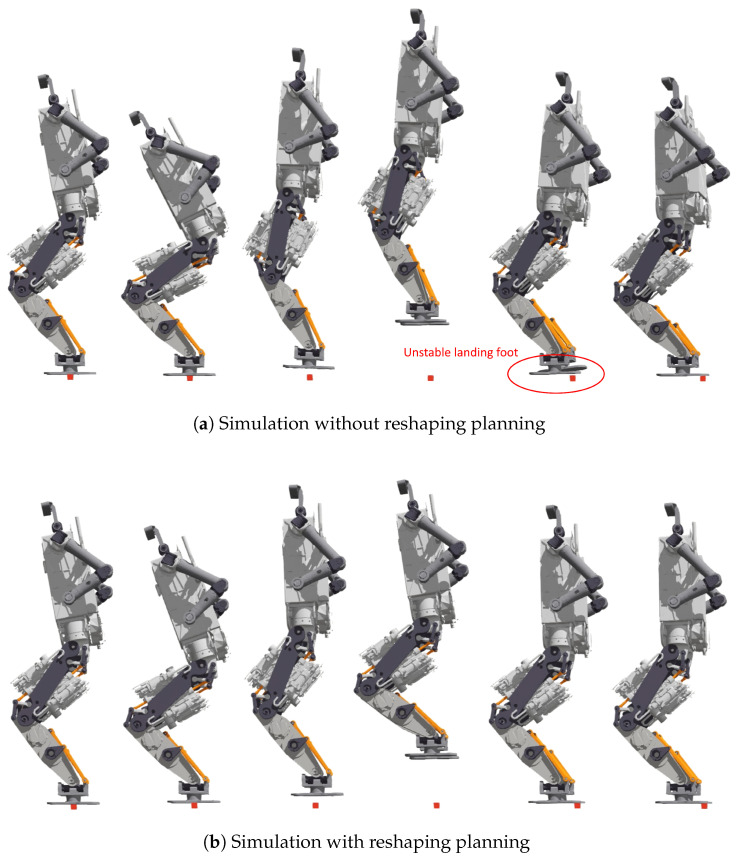
Simulation shots: effect of the reshaping module. (**a**): without reshaping planning. (**b**): with reshaping planning.

**Figure 6:**
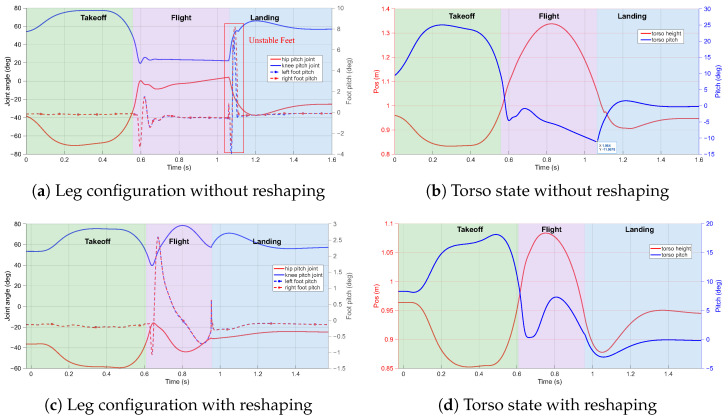
Analysis: leg coordination and torso regulation. (**a**,**b**) Without reshaping planning. (**c**,**d**) With reshaping planning.

To evaluate the role of the reshaping module in in-place jumping, comparative simulations were conducted with and without the proposed module enabled. Both cases shared the same offline-optimized jumping trajectory as the initial reference, ensuring that the observed performance differences were solely attributed to the presence of reshaping planning. All simulations were performed in the Webots physics-based simulation environment [32,33]. When the reshaping module is disabled, the robot executes the offline reference trajectory in an open-loop manner. As shown in Figure 5a and Figure 6a,b, the hip-pitch and knee-pitch joint angles remain nearly constant during the flight phase, indicating that the lower limbs are not actively utilized for mid-air posture regulation. In addition, the robot exhibits a longer flight duration in the offline case, which implies a higher required takeoff velocity to achieve the same jumping height.

Consequently, deviations introduced during takeoff cannot be compensated once ground contact is lost, and the increased takeoff velocity further imposes higher dynamic tracking demands on the joints. This limitation is clearly reflected in the torso motion: during the flight phase, the torso exhibits a pronounced backward rotation, reaching a maximum deviation of approximately $-11^{\circ }$, as illustrated in Figure 6b. This behavior reveals insufficient regulation of angular momentum about the center of mass. Since no effective leg reconfiguration occurs in mid-air, the attitude error persists and directly affects the subsequent landing configuration. As shown in Figure 5a and Figure 6a,b, the left and right foot pitch angles diverge noticeably during landing, leading to asymmetric ground contact and partial foot landing, which ultimately results in an unstable landing posture. Such characteristics suggest that the offline strategy may be particularly challenging to realize on real hardware, where actuator bandwidth and tracking accuracy are inherently limited.

To further quantify the contribution of the reshaping module, Table 5 compares representative performance metrics between the offline-only case and the proposed reshaping case. The takeoff, flight, and landing phases were identified from the foot touchdown reference signal. Tracking errors were evaluated over the plotted motion window, while landing posture and contact-quality metrics were evaluated after touchdown until the end of the landing window. The peak impact force was computed as the maximum total vertical ground-reaction force within the first 0.2 s after touchdown. The force asymmetry at peak impact is defined as

(47) $$\eta_{F}=\frac{|F_{z,L}-F_{z,R}|}{F_{z,L}+F_{z,R}},$$

where $F_{z,L}$ and $F_{z,R}$ denote the vertical ground-reaction forces of the left and right feet, respectively.

As shown in Table 5, the reshaping module substantially improves both tracking performance and landing robustness. Compared with the offline-only case, the torso pitch RMSE decreases from $6.59^{\circ }$ to $1.83^{\circ }$, corresponding to a 72.3% reduction. At touchdown, the torso pitch deviation is reduced from $11.07^{\circ }$ to $0.91^{\circ }$, indicating that the robot enters the landing phase with a much more favorable trunk attitude. After touchdown, the maximum torso pitch deviation and pitch rate are reduced by 72.5% and 63.9%, respectively.

The improvement is also evident in the foot-contact behavior. Without reshaping, the maximum foot pitch after touchdown reaches $8.20^{\circ }$, and the maximum left–right foot pitch difference is $4.85^{\circ }$, indicating asymmetric and unstable foot contact. With the reshaping module, these two values decrease to $0.31^{\circ }$ and $0.11^{\circ }$, respectively, suggesting nearly symmetric full-foot landing. Moreover, the peak total vertical impact force decreases from 12.79kN to 10.44kN, while the force asymmetry at peak impact is reduced from 0.942 to 0.079. These quantitative results demonstrate that the proposed reshaping strategy improves landing posture regulation, contact symmetry, and impact mitigation without requiring online trajectory re-optimization.

### 4.3. Hardware Jumping Experiments

**Figure 7:**
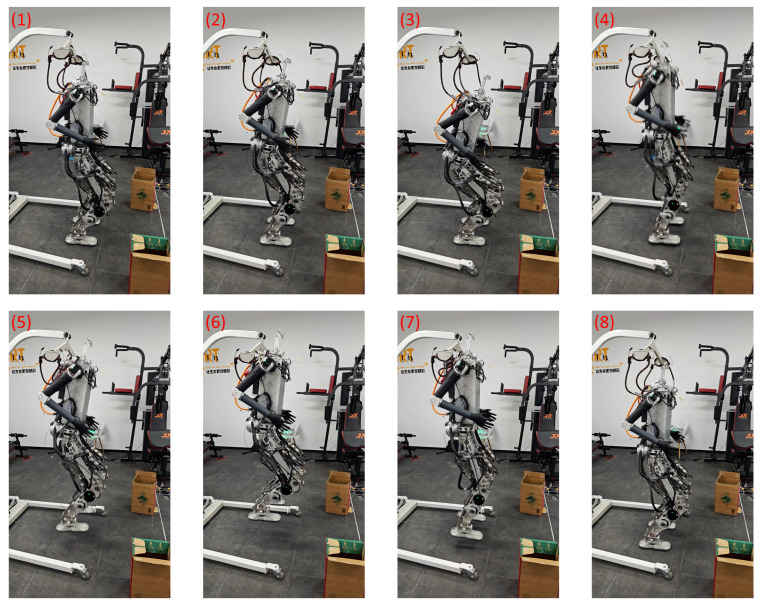
Snapshots of the complete in-place jumping process of the real robot: (1)–(8) show the sequential snapshots from takeoff preparation to stable landing.

**Figure 8:**
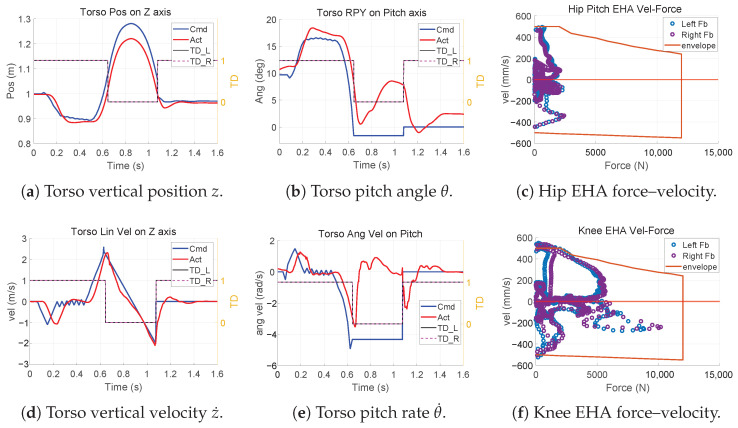
Physical jumping experiment results.

The in-place jumping experiments conducted on a full-sized humanoid robot further validate the effectiveness of the proposed reshaping planning and whole-body control framework under realistic physical constraints. As shown in Figure 7, the robot completes a stable sequence of takeoff, flight, and landing without external support, achieving an in-place jumping height of approximately 30cm. The quantitative results in Figure 8 indicate that the torso vertical position and velocity profiles exhibit smooth lift-off and deceleration characteristics, demonstrating that the system is capable of generating and absorbing momentum in a continuous and well-regulated manner during highly dynamic motions.

In addition to vertical motion performance, effective trunk posture regulation is maintained throughout the jumping motion. As shown in Figure 8b,e, both the torso pitch angle and pitch rate remain within bounded ranges during takeoff, flight, and landing, with no abrupt transitions or high-frequency oscillations observed near touchdown. These results indicate that the reshaping module can reshape the reference trajectory online based on the estimated system state, enabling the whole-body controller to regulate trunk posture reliably in the presence of model uncertainties, actuator non-idealities, and contact impacts.

From an actuation perspective, the physical feasibility of the jumping motion is further confirmed. As shown in Figure 8c,f, the force–velocity operating points of the hip and knee electro-hydrostatic actuators (EHAs) remain mostly within their feasible envelopes throughout the experiments, with only brief excursions near the boundaries and no observable actuator saturation. The measured joint-level performance summarized in Table 6 and Table 7 shows that the required stroke ranges, velocities, forces, and power demands of both EHA-driven and motor-driven joints are compatible with the hardware limits. These results demonstrate that the proposed control and planning framework can achieve dynamic in-place jumping of approximately 30cm while strictly respecting actuator constraints, thereby confirming its practicality and engineering applicability on full-sized humanoid robots. Moreover, the robot was able to perform repeated in-place jumping motions using the same control and planning framework, with a near-100% success rate observed across trials. In the reported hardware tests, the robot completed 10 successful jumps out of 10 trials, where a trial was considered successful if the robot achieved liftoff, recovered double-foot contact, and remained standing for at least 3 s after touchdown. This repeatability further indicates that the proposed framework provides sufficient robustness margins for executing highly dynamic motions on full-sized humanoid robots. It should be noted that the robustness discussed in this work mainly refers to execution robustness against modeling errors, actuation non-idealities, takeoff impulse mismatch, and landing-state deviations, rather than complete robustness against arbitrary external disturbances. In practice, the simulation model and the physical robot inevitably differ in total mass, mass distribution, and actuator response. Nevertheless, the proposed framework can still be transferred to the physical robot and achieve stable in-place jumping and landing recovery. As a preliminary supplementary test, we also repeated the jump with an additional payload of approximately 5kg using the same control framework, and stable landing recovery was observed. Since this test was not designed as a systematic payload-robustness evaluation and only limited trials were conducted, it is reported here only as qualitative supplementary evidence. Systematic push-disturbance and payload-variation tests will be investigated in future work.

## 5. Conclusions

This paper presented an offline-to-online planning and whole-body control framework for dynamic bipedal jumping and validated it on a full-sized humanoid robot. By combining phase-consistent trajectory optimization with execution-time adaptation and constraint-aware whole-body control, the proposed approach enables stable in-place jumping on real hardware.

Compared with recent learning-based jumping and parkour approaches [7,9,10], the proposed framework avoids end-to-end policy training and large-scale sim-to-real transfer, and instead follows a model-based planning-control pipeline for controlled validation on heavy full-sized humanoid hardware. Compared with online trajectory-optimization or MPC-based jumping methods [13,22,25], it uses low-dimensional online reference reshaping rather than high-dimensional online re-optimization, improving real-time performance and hardware executability while preserving the offline trajectory structure. Unlike trajectory-optimization methods that mainly focus on offline feasible motion generation, this work emphasizes the execution of optimized jumping references on a heavy full-sized humanoid robot by integrating lightweight online reshaping, whole-body control, and EHA-level actuator mapping.

Experimental results show that, although noticeable discrepancies exist between planned and executed takeoff motions, the overall jumping behavior remains stable and repeatable. This indicates that robust bipedal jumping does not rely on precise realization of offline plans, but rather on systematic integration of planning, online adaptation, and whole-body control.

Future work will extend the framework to more challenging jumping tasks, including forward and directional jumping, consecutive jumping, and terrain-adaptive takeoff and landing.

## Figures and Tables

**Table 1:** Comparison with Related Jumping-Control Frameworks.

Method Category	Online Re-Opt.	Dynamics Representation	Landing Adaptation	Main Limitation
Reduced-order	Optional	Simplified model	Limited	Limited model fidelity for highly dynamic full-body jumping
MPC-based	Yes	Partial/Full	Yes	High computational cost caused by online optimization
RL-based	No	Learned implicitly	Implicit	Sim-to-real transfer challenges and limited interpretability
Proposed method	No	Full-body dynamics via WBC	Online reshaping	Requires platform-specific tuning of reshaping parameters

**Table 2:** Task and Constraint Configuration of the WBC for Jumping.

Task	Enabled	Constraint	Enabled
CoM trajectory		Floating-base dynamics	✓
Trunk position	✓	Foot contact wrench	✓
Trunk orientation	✓	Joint torque limits	✓
Foot trajectory	✓	Joint position limits	✓
Foot wrench		Joint power limits	✓

**Table 3:** Main Physical Parameters of the Robot.

Dimensions and Mass Properties					
Total mass *m*	Height *h*	Head mass	Trunk mass	Arm mass (both)	Leg mass (both)
79.5 [kg]	1.77 [m]	0.9 [kg]	33.5 [kg]	4.6 [kg]	37.7 [kg]
Leg Joint Motion Ranges and Peak Actuator Outputs					
Hip Yaw	Hip Roll	Hip Pitch	Knee Pitch	Ankle Pitch	Ankle Roll
−50°∼90°	−20°∼45°	−10°∼70°	80°∼160°	−20°∼65°	−25°∼25°
140 [rpm]	126 [rpm]	36 [rpm]	36 [rpm]	140 [rpm]	140 [rpm]
90 [Nm]	300 [Nm]	12,000 [N]	12,000 [N]	90 [Nm]	90 [Nm]

**Table 4:** Planning and Control Parameters.

Group	Parameters	Value
Trajectory optimization Section 2.1	Step time	50 ms
	Horizon per phase	30
	Min. CoM height	0.40m
	Takeoff and Land time	1 s
	Flight time	0.4∼0.6 s
Online Reshaping Section 2.3	$k_{v}$	[0.1,0.1]
	$k_{h}$	[0.2,0.1]
	$a_{x},a_{z},a_{\mathrm{pitch}}$	[1.0,−2.0,2.0]

**Table 5:** Quantitative comparison of jumping performance with and without the reshaping module.

Metric	Without Reshaping	With Reshaping	Reduction (%)
Phase and tracking performance			
Flight duration (s)	0.506	0.350	–
Torso height RMSE (m)	0.051	0.045	11.3
Torso pitch RMSE (deg)	6.59	1.83	72.3
Landing posture and contact quality			
Touchdown torso pitch (deg)	11.07	0.91	91.8
Max. post-TD torso pitch (deg)	11.07	3.04	72.5
Max. post-TD pitch rate (deg/s)	250.3	90.3	63.9
Max. post-TD foot pitch (deg)	8.20	0.31	96.2
Max. L–R foot pitch diff. (deg)	4.85	0.11	97.7
Impact-force response			
Peak vertical impact force (kN)	12.79	10.44	18.4
Force asymmetry at impact peak	0.942	0.079	91.6

**Table 6:** Measured EHA Joint Performance During In-Place Jumping.

Electro-Hydrostatic Actuator (EHA) Joints				
**Joint**	**Stroke** **Range (mm)**	**Max. Velocity** **(mm/s)**	**Max. Force** **(N)**	**Max. Power** **(W)**
Hip-pitch EHA	287–325	500	2400	620
Knee EHA	278–322	550	10,200	1640

**Table 7:** Electric Joint Performance During In-Place Jumping.

Electric Motor-Driven Joints			
**Joint**	**Max. Speed** **(rad/s)**	**Max. Torque** **(Nm)**	**Max. Power (W)** **(pos./neg.)**
Hip roll	1.2	64	30/−40
Hip yaw	1.4	14	10/−10
Ankle pitch	8.1	98	410/−340
Ankle roll	7.6	87	420/−380

## Data Availability

The data presented in this study are available on reasonable request from the corresponding authors.

## Appendix A. Geometric Mapping of the EHA-Driven Four-Bar Mechanism

This appendix details the geometric derivation of the nonlinear mapping between joint-space variables and actuator-space quantities for the electro-hydrostatic actuator (EHA)-driven four-bar mechanisms used in the hip-pitch and knee joints. These results are omitted from the main text for brevity but are included here to ensure completeness and reproducibility.

### Appendix A.1. Forward Kinematics

The geometry of the EHA-driven four-bar linkage is shown in Figure 3. Links AB, BC, AO, CO, and CP have fixed lengths. Points *O*, *C*, and *P* are rigidly connected, implying a constant angle ∠OCP. The segment BP has variable length and corresponds to the EHA actuator stroke *L*. The driven revolute joint is located at point *O*, with joint angle defined as q=∠AOC.

Given the actuator stroke *L*, the joint angle *q* is determined by the geometric constraints of the linkage. Applying the law of cosines yields

(A1) $$\begin{array}{cc} \theta_{BCP} & =\arccos\;\left(\frac{L_{BC}^{2}+L_{CP}^{2}-L^{2}}{2L_{BC}L_{CP}}\right),\\ \theta_{BCO} & =2\pi -\theta_{BCP}-\theta_{PCO},\\ L_{BO} & =\sqrt{L_{BC}^{2}+L_{OC}^{2}-2L_{BC}L_{OC}\cos\;\left(\theta_{BCO}\right)},\\ \theta_{BOC} & =\arccos\;\left(\frac{L_{OB}^{2}+L_{OC}^{2}-L_{BC}^{2}}{2L_{OB}L_{OC}}\right),\\ \theta_{AOB} & =\arccos\;\left(\frac{L_{OB}^{2}+L_{OA}^{2}-L_{AB}^{2}}{2L_{OB}L_{OA}}\right),\\ q & =\theta_{BOC}+\theta_{AOB}. \end{array}$$

These expressions define a nonlinear forward kinematic mapping q=f(L).

### Appendix A.2. Velocity and Force Transmission

Differentiating the forward kinematics yields the velocity relationship

(A2) $$\dot{q}=J\left(L\right)\dot{L},$$

where J(L) is the configuration-dependent transmission Jacobian. Assuming ideal power transmission, the actuator force *F* and joint torque τ satisfy

(A3) $$F\dot{L}=\tau \dot{q},$$

which leads to

(A4) $$\tau =J^{-1}\left(L\right)F.$$

Thus, both joint velocity and torque are modulated by the nonlinear transmission characteristics of the four-bar mechanism.

### Appendix A.3. Inverse Kinematics

Given a desired joint angle *q*, the corresponding actuator stroke *L* can be recovered from the same geometric constraints. The intermediate quantities are computed as

(A5) $$\begin{array}{cc} L_{AC} & =\sqrt{L_{OA}^{2}+L_{OC}^{2}-2L_{OA}L_{OC}\cos q},\\ \theta_{ACO} & =\arccos\;\left(\frac{L_{AC}^{2}+L_{OC}^{2}-L_{OA}^{2}}{2L_{AC}L_{OC}}\right),\\ \theta_{ACB} & =\arccos\;\left(\frac{L_{AC}^{2}+L_{BC}^{2}-L_{AB}^{2}}{2L_{AC}L_{BC}}\right),\\ \theta_{BCP} & =2\pi -\theta_{ACO}-\theta_{ACB}-\theta_{PCO},\\ L & =\sqrt{L_{BC}^{2}+L_{CP}^{2}-2L_{BC}L_{CP}\cos\;\left(\theta_{BCP}\right)}. \end{array}$$

This inverse mapping converts joint-space references into actuator-space commands for the EHA-driven joints.

### Appendix A.4. Discussion

The four-bar mechanism introduces a configuration-dependent nonlinear transmission between actuator force, actuator velocity, and joint torque. During highly dynamic motions such as jumping, takeoff, and landing, this coupling can substantially constrain the realizable joint-space commands, even when nominal joint-level limits are satisfied. The mappings derived in this appendix are therefore essential for actuator-aware execution and for interpreting the actuator-level performance reported in the experimental results.
